# Antisense lncRNA PCNA-AS1 promotes esophageal squamous cell carcinoma progression through the miR-2467-3p/PCNA axis

**DOI:** 10.1515/med-2022-0552

**Published:** 2022-09-20

**Authors:** Tao Hu, Yunfeng Niu, Jianfeng Fu, Zhiming Dong, Dongwei He, Junfeng Liu

**Affiliations:** Department of Anesthesiology, The Fourth Hospital of Hebei Medical University, Shijiazhuang, Hebei, China; Laboratory of Pathology, Hebei Cancer Institute, The Fourth Hospital of Hebei Medical University, Shijiazhuang, Hebei, China; Department of Thoracic Surgery, The Fourth Hospital of Hebei Medical University, Shijiazhuang, Hebei, China

**Keywords:** PCNA-AS1, PCNA, miR-2467-3p, ESCC

## Abstract

Multiple studies have indicated that long non-coding RNAs are aberrantly expressed in cancers and are pivotal in developing various tumors. No studies have investigated the expression and function of long non-coding antisense RNA PCNA-AS1 in esophageal squamous cell carcinoma (ESCC). In this study, the expression of PCNA-AS1 was identified by qRT–PCR. Cell function assays were used to explore the potential effect of PCNA-AS1 on ESCC progression. A prediction website was utilized to discover the relationships among PCNA-AS1, miR-2467-3p and proliferating cell nuclear antigen (PCNA). Dual luciferase reporter gene and RNA immunoprecipitation (RIP) assays were executed to verify the binding activity between PCNA-AS1, miR-2467-3p and PCNA. As a result, PCNA-AS1 was highly expressed in ESCC and was associated with patient prognosis. PCNA-AS1 overexpression strongly contributed to ESCC cell proliferation, invasion and migration. PCNA-AS1 and PCNA were positively correlated in ESCC. Bioinformatics analysis, RIP and luciferase reporter gene assays revealed that PCNA-AS1 could act as a competitive endogenous RNA to sponge miR-2467-3p, thus upregulating PCNA. In conclusion, the current outcome demonstrates that PCNA-AS1 may be a star molecule in the treatment of ESCC.

## 1 Introduction

Esophageal cancer is the sixth most typical carcinoma-related fatality worldwide [1]. Esophageal squamous cell carcinoma (ESCC) and esophageal adenocarcinoma are the main histological forms of esophageal cancer [2]. The morbidity and mortality rates of ESCC in China vary across different regions, such as the Hebei, Henan and Shanxi provinces [3]. In spite of significant progress in the diagnosis and treatment of ESCC, the 5-year survivorship of patients is still very weak [4]. In addition, most ESCC patients are diagnosed in the medium to late stage [5]. Therefore, the molecular markers for early diagnostics and prognostic evaluation are critical to increasing the survival rate of ESCC patients.

Long non-coding RNA (lncRNA), “an RNA molecule more than 200 nucleotides in length with a conserved secondary structure,” has no ability to encode proteins [6]. LncRNAs engage with proteins, DNA and RNA to regulate gene expression via various mechanisms [7]. LncRNAs have been reported to be localized in both the nucleus and cytoplasm. LncRNAs exert their functions based on subcellular localization. Cytoplasmic lncRNAs can bind and “sponge” miRNAs, thereby preventing miRNA binding to target mRNAs [8]. In the nucleus, lncRNAs regulate gene transcription, chromatin modification and mRNA stability [9]. LncRNAs play critical functions in carcinogenesis, including cell cycle, endothelial-mesenchymal transition, invasion and migration [10]. Most research has indicated that lncRNAs are variably expressed in various cancers, including ESCC [11].

The purpose of this study was to establish the critical biological functions of PCNA-AS1 in ESCC. We discovered that proliferating cell nuclear antigen (PCNA) antisense RNA1 (PCNA-AS1) was intensely expressed in ESCC and facilitated cell proliferation and metastasis *in vitro*. In this study, the interaction mechanism between PCNA-AS1 and PCNA was further investigated, thus providing new ESCC prognosis and treatment strategies.

## 2 Materials and methods

### 2.1 Bioinformatics analysis

The esophageal cancer chip GSE75241 from the GEO database (https://www.ncbi.nlm.nih.gov/geo/) was adopted to determine the variably expressed genes among ESCC and normal tissues (log|fold change| > 1, *P* < 0.05). miRBase (http://www.mirbase.org/) and TargetScan (http://www.targetscan.org/) were used to determine miRNAs that bind to PCNA-AS1 and PCNA 3′-UTR.

### 2.2 Patients and specimens

Sixty specimen pairs of tumor and non-tumor tissues were obtained from ESCC surgery patients at the Fourth Hospital of Hebei Medical University from 2015 to 2016 (Table S1). These ESCC patients did not undergo chemotherapy or radiotherapy treatments before the operation. All ESCC tissue specimens were confirmed by HE staining, and no cancerous invasion was detected in the normal tissues. The ethics committee endorsed this study, and all patients who provided clinical samples signed an informed consent form.

### 2.3 Cell culture

Human ESCC cell lines (Eca109, Kyse150, Yes2, TE13 and TE1) were sourced from American Type Culture Collection. Cells were cultured in RPMI 1640 medium containing 10% fetal bovine serum (FBS) and placed in a 5% CO_2_ incubator.

### 2.4 RNA isolation and quantitative real-time PCR (qRT-PCR) assay

Under the reagent protocol, total RNA was isolated from ESCC cells and tissues with TRIzol (Invitrogen) and reverse transcribed to cDNA with the transcriptor first-strand cDNA synthesis kit (Roche). The qRT-PCR assay was determined using the StepOne Real-Time fluorescent quantitative PCR system (Applied Biosystems) and GoTaq^®^ qPCR Master Mix (Promega). The endogenous controls for RNA were GAPDH and U6. 2^−ΔΔCT^ indicated the expression of RNA. The primers employed are shown in Table S2.

### 2.5 Plasmids, shRNAs, mimics, inhibitors

The shRNA targeting PCNA-AS1 and the pcDNA3.1-PCNA-AS1 plasmid were obtained on GenScript. The miR-2467-3p mimics, inhibitors and controls were purchased from Gene Pharma (Tables S3 and S4). Cell transfection assay was performed using Lipofectamine 2000 reagent.

### 2.6 Cell proliferation

Cell proliferation was undertaken with the MTS and clone formation assays. For the MTS assay, 1 × 10^3^ transfected cells were seeded into each well of a 96-well plate. After 0, 24, 48, 72 and 96 h, 20 µL MTS (500 µg/mL) was added to each well and continued to incubate for 2 h. The absorbance value at 490 nm was calculated with a microplate reader. For the clone formation assay, 1 × 10^5^ transfected cells were grown in a 6-well plate and sustained for 1 week. Clones of over 50 cells were counted as one clone.

### 2.7 Migration and invasion assays

Cell migration and invasion were investigated in the absence of Matrigel (migration) or in the presence of Matrigel (invasion) using a membrane with 8 μm (Corning). A total of 5 × 10^4^ cells were added to the upper chamber, and 500 μL of 10% FBS medium was placed in the bottom chamber. About 50 μL Matrigel (BD) was applied to the membrane surface for invasion analysis. After 24 h of incubation, invading cells were immobilized with neutral formaldehyde and incubated with crystal violet staining for 20 min. The cells were enumerated by inverted microscopy.

### 2.8 Cell cycle and apoptosis assays

Transfected cells were incubated for 48 h, and then pre-cooled 70% alcohol was added at 4°C overnight. The cells were incubated with propidium iodide for 30 min at 4°C. Finally, cell cycles were analyzed and determined by a flow cytometer (FC500 type flow cytometer; Beckman Coulter). The apoptosis assay was used to analyze cells with the PE Apoptosis Kit (BD) according to the manufacturer’s instructions.

### 2.9 Western blot assay

ESCC cells were treated in RIPA lysis buffer containing PMSF (Solarbio). Samples were detached using 10% SDS gel and transferred to a PVDF membrane. The membranes were soaked in 5% milk for 1 h, followed by incubation with primary antibodies at 4°C overnight: PCNA (Bioss, China, catalog no. bs-0754R; 1:1,000) and ACTB (Bioworld, USA, catalog no. BS1002; 1:1,000). After rinsing with TBST, the membranes were placed in the secondary antibody for 1 h at 37℃. The signal was measured and visualized with enhanced chemiluminescence detection reagents.

### 2.10 Dual luciferase reporter gene assay

We cotransfected luciferase reporter vectors (pGLO-PCNA-AS1-1 (WT), pGLO-PCNA-AS1-1 (MUT), pGLO-PCNA-AS1-2 (WT) or pGLO-PCNA-AS1-2 (MUT)) with miR-2467-3p mimics or miR-NC into Eca109 cells with Lipofectamine 2000. After 48 h of incubation, the fluorescence activity was detected using a luciferase assay kit (Promega) according to the manufacturer’s guidelines.

### 2.11 RNA immunoprecipitation (RIP) assay

The constructed plasmids pcDNA3.1-MS2 and pcDNA3.1-PCNA-AS1-MS2 were cotransfected into Eca109 cells with pMS2-GFP. After transfected cells were cultured for 48 h, the cells were collected and used for RIP experiments with Magna RIP™ Kit and GFP antibody. Total RNA was purified by Trizol, and then miR-2467-3p expression was analyzed by qRT-PCR following the reagent protocol.

### 2.12 Statistical analysis

Data are shown as mean ± SD. Group comparisons were performed by one-way ANOVA or *t*-test. The correlation was assessed by Spearman’s method. *P* < 0.05 was regarded as statistically different.

## 3 Results

### 3.1 PCNA-AS1 is highly expressed in ESCC and associated with poor prognosis of ESCC patients

**Figure 1:**
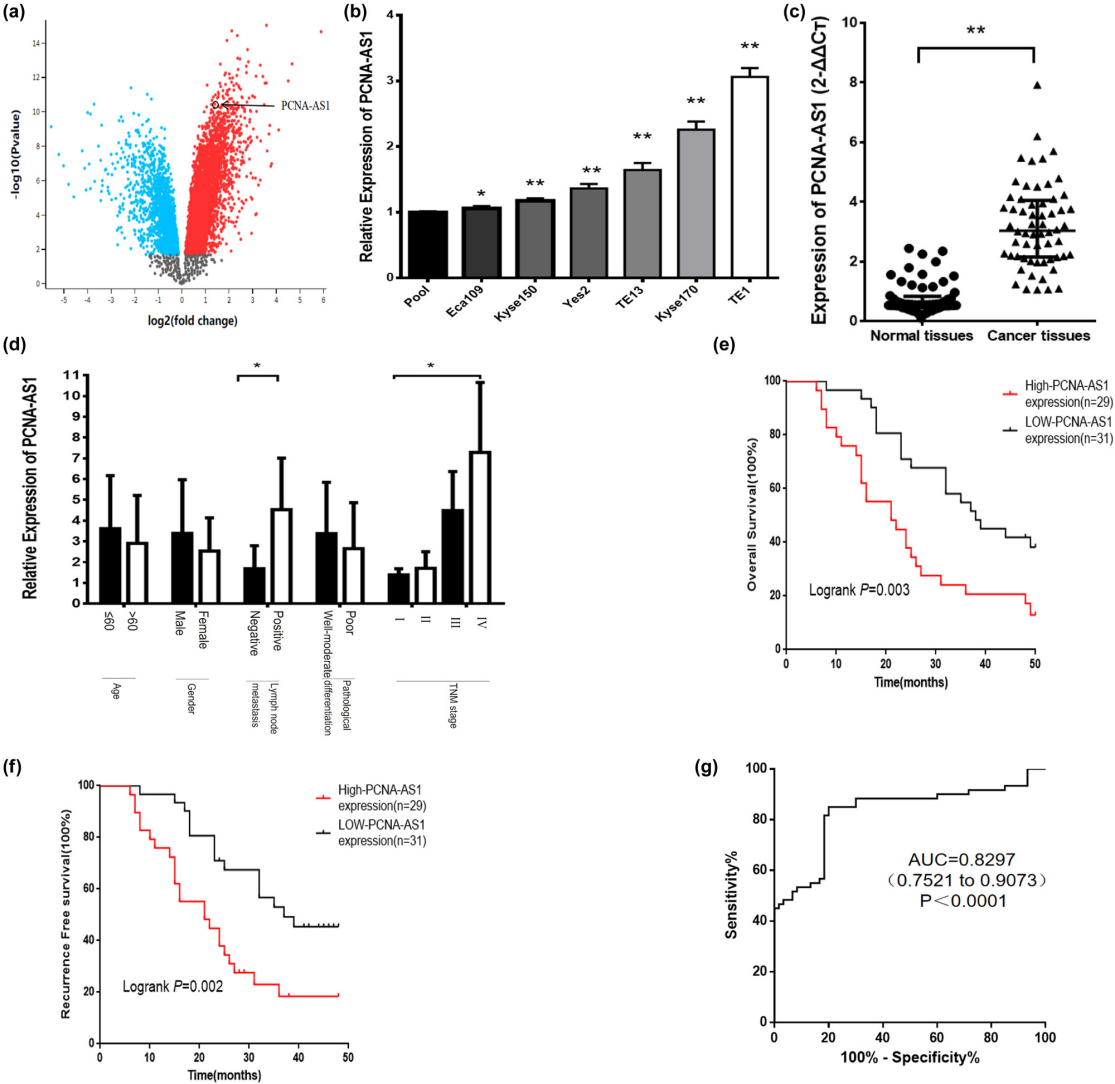
PCNA-AS1 is significantly upregulated and associated with clinicopathological characteristics. (a) PCNA-AS1 is highly expressed in GSE75241 (log|fold change| > 1, *P* < 0.05). (b) The expression of PCNA-AS1 in a panel of human esophageal cancer cell lines by qRT-PCR. (c) The expression of PCNA-AS1 in 60 pairs of ESCC tissues and normal tissues. (d) The relationship between PCNA-AS1 expression and clinicopathological characteristics of ESCC patients. (e and f) Expression of PCNA-AS1 was negatively associated with the OS and RFS of patients with ESCC. (g) ROC curve analysis shows an AUC of 0.8297 to distinguish ESCC from adjacent normal tissues. *P < 0.05 and **P < 0.01.

We used the GSE75241 dataset to examine differentially expressed genes in ESCC compared to non-tumor tissues and identified 2,042 differentially expressed genes, including 496 downregulated and 1,056 upregulated genes. One of the differentially expressed genes, PCNA-AS1, was highly expressed in ESCC (Figure 1a). In the meantime, we examined PCNA-AS1 in human esophageal cancer cell lines and discovered that PCNA-AS1 was highly expressed in Kyse170 and TE1 cells (Figure 1b). Moreover, PCNA-AS1 was obviously upregulated in ESCC tissues (*P* < 0.05, Figure 1c).

We next explored the correlation between PCNA-AS1 expression and ESCC patient profiles. High expression of PCNA-AS1 was remarkably correlated with lymph node metastasis and TNM stage (both *P* < 0.01, Figure 1d). Moreover, we divided them into two groups based on the median of PCNA-AS1 expression. ESCC patients with high PCNA-AS1 expression showed considerably lower overall survival (OS) and recurrence-free survival (RFS) than those with low expression (both *P* < 0.01, Figure 1e and f). The receiver operating characteristic (ROC) curve analysis indicated that the area under the curve (AUC) of PCNA-AS1 was 0.8297 (0.7521–0.9073) (Figure 1g).

### 3.2 PCNA-AS1 contributes to the malignant biological behavior of ESCC cells

**Figure 2:**
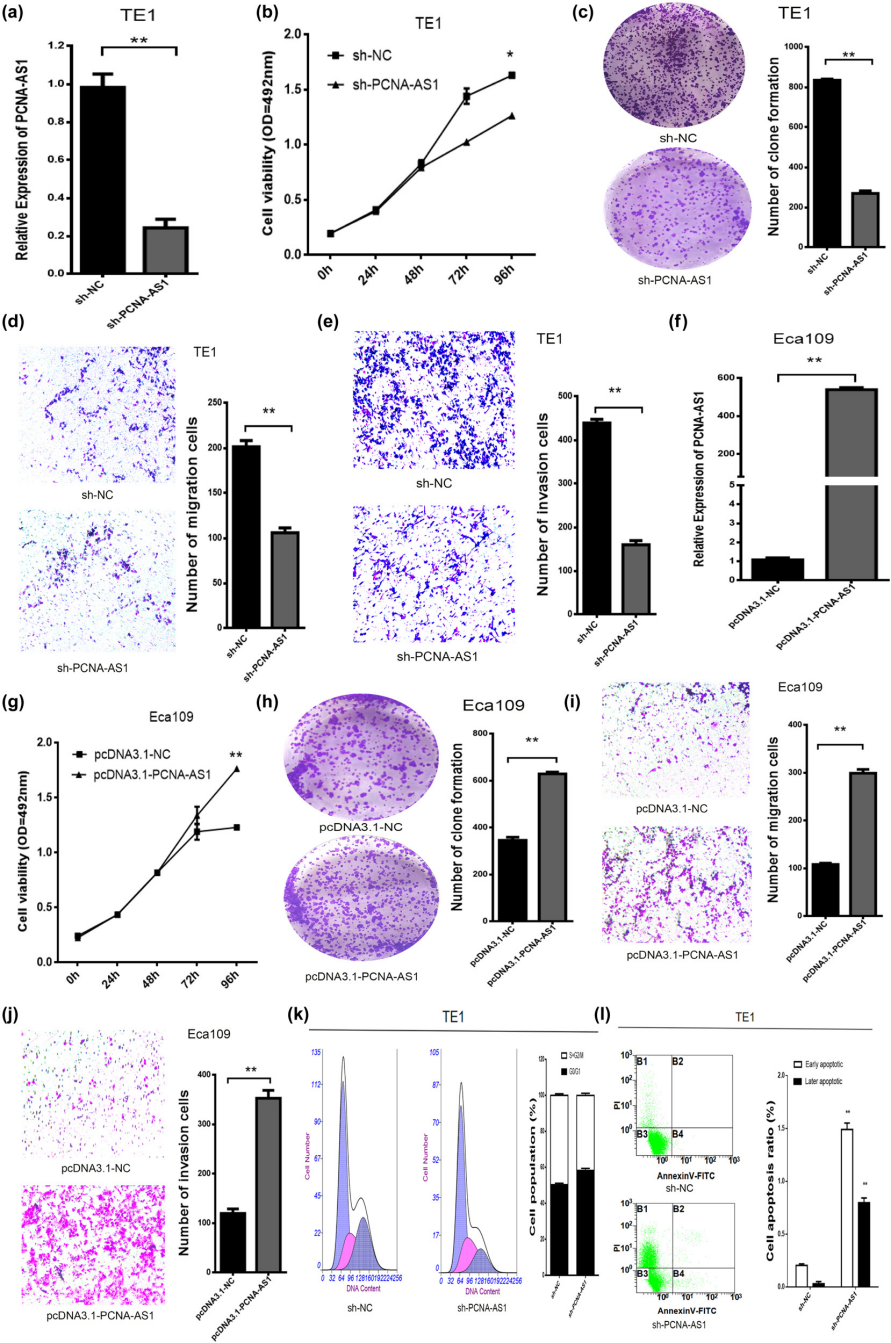
The effect of PCNA-AS1 on the biological behavior of ESCC cells. (a–e) Knockdown of PCNA-AS1 on cell proliferation, migration, clone information and invasion. (f–j) Overexpression of PCNA-AS1 on cell proliferation, migration, clone information and invasion. (k and l) Knockdown of PCNA-AS1 on cell cycle and apoptosis. *P < 0.05 and **P < 0.01.

To determine the biological function of PCNA-AS1, we performed gain- and loss-of-function assays in ESCC cells using plasmid overexpression or shRNA-mediated downregulation. The qRT-PCR results confirmed that shRNA targeting PCNA-AS1 (sh-PCNA-AS1) substantially suppressed PCNA-AS1 in TE1 cells (Figure 2a). MTS assays showed that PCNA-AS1 knockdown markedly suppressed the cell proliferation of TE1 cells (*P* < 0.01, Figure 2b). A similar growth-inhibitory effect was observed with clone formation assays (Figure 2c). We further revealed that the migration and invasion abilities of TE1 cells were significantly inhibited after transfection with sh-PCNA-AS1 (Figure 2d and e).

We also upregulated PCNA-AS1 expression in Eca109 cells using the pcDNA3.1-PCNA-AS1 overexpression plasmid and confirmed the upregulated expression by qRT-PCR (Figure 2f). Overexpression of PCNA-AS1 markedly enhanced the cell proliferation, migration and invasion abilities of Eca109 cells (Figure 2g–j). In addition, flow cytometry analysis revealed that the knockdown of PCNA-AS1 resulted in a remarkable induction of G0/G1 phase arrest (Figure 2k). Apoptosis assays revealed increased numbers of early and late apoptotic cells in TE1 cells with the knockdown of PCNA-AS1 (Figure 2l).

### 3.3 PCNA is overexpressed in ESCC and positively correlates with the expression of PCNA-AS1

**Figure 3:**
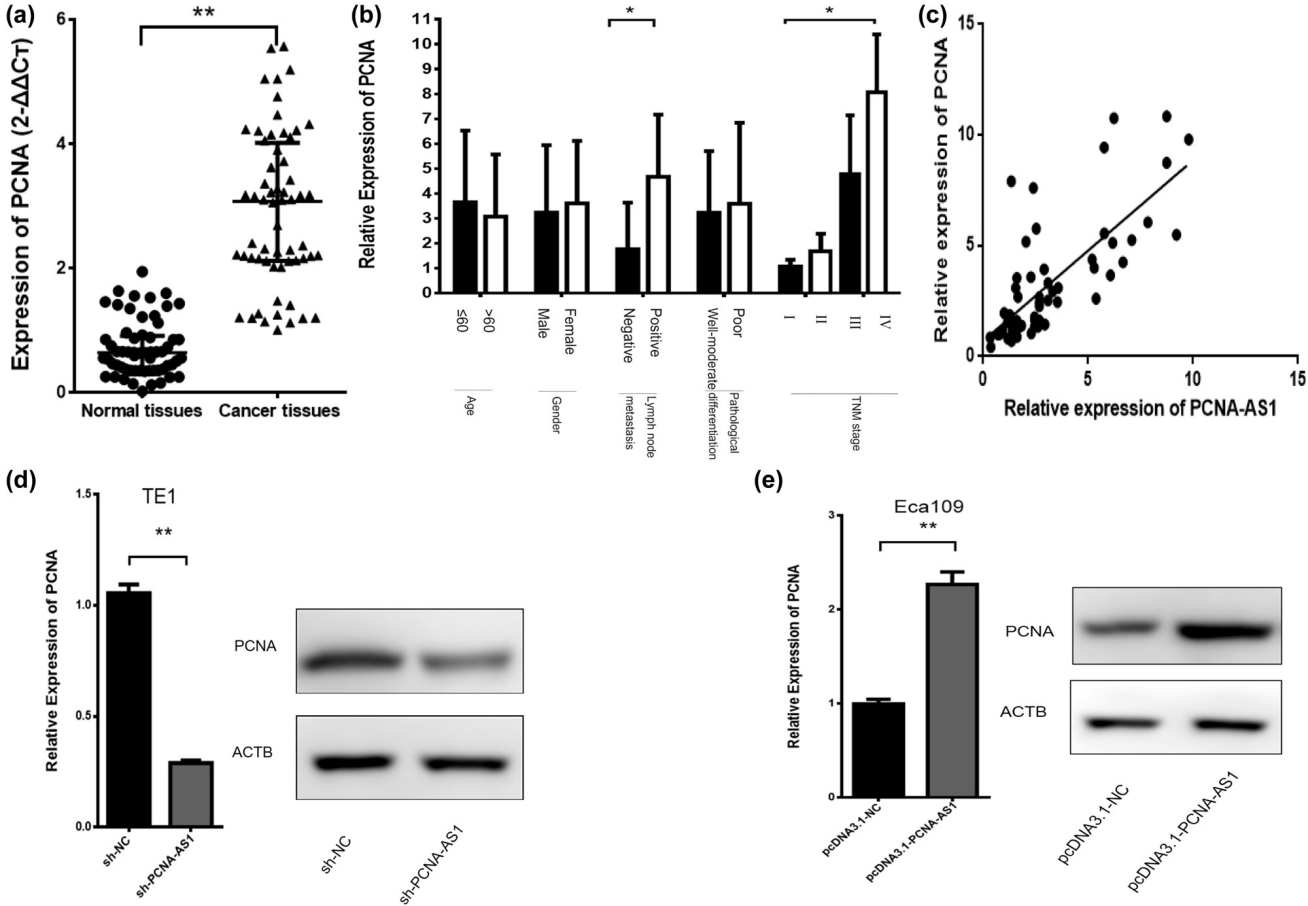
PCNA acts as an oncogene in ESCC and is regulated by PCNA-AS1. (a) The expression of PCNA in 60 pairs of ESCC tissues and normal tissues by the qRT-PCR assay. (b) The relationship between PCNA expression and the clinicopathological characteristics of ESCC patients. (c) The relationship between PCNA-AS1 and PCNA in ESCC (*r* ^2^ = 0.552, *P* < 0.01). (d) Knockdown of PCNA-AS1 significantly suppressed PCNA expression levels in TE1 cells. (e) Overexpression of PCNA-AS1 significantly promoted PCNA expression levels in Eca109 cells. *P < 0.05 and **P < 0.01.

As PCNA-AS1 is a long non-coding antisense RNA of PCNA, to examine the association between PCNA-AS1 and PCNA in ESCC, we first assessed the expression pattern of PCNA in ESCC tissues. PCNA mRNA was upregulated in ESCC tissues (Figure 3a). In addition, the expression level of PCNA was appreciably correlated with lymph node metastasis and the TNM stage of ESCC patients (Figure 3b). Further study showed that PCNA exhibited a concordant co-regulation with PCNA-AS (*r* ^*2*^ = 0.552, *P* < 0.01, Figure 3c).

We further explored the influence of PCNA-AS1 on PCNA expression. It was found that the knockdown of PCNA-AS1 meaningfully decreased PCNA expression in TE1 cells (*P* < 0.01, Figure 3d). Overexpression of PCNA-AS1 essentially promoted PCNA expression in Eca109 cells (*P* < 0.01, Figure 3e).

### 3.4 miR-2467-3p is a direct target of PCNA-AS1

**Figure 4:**
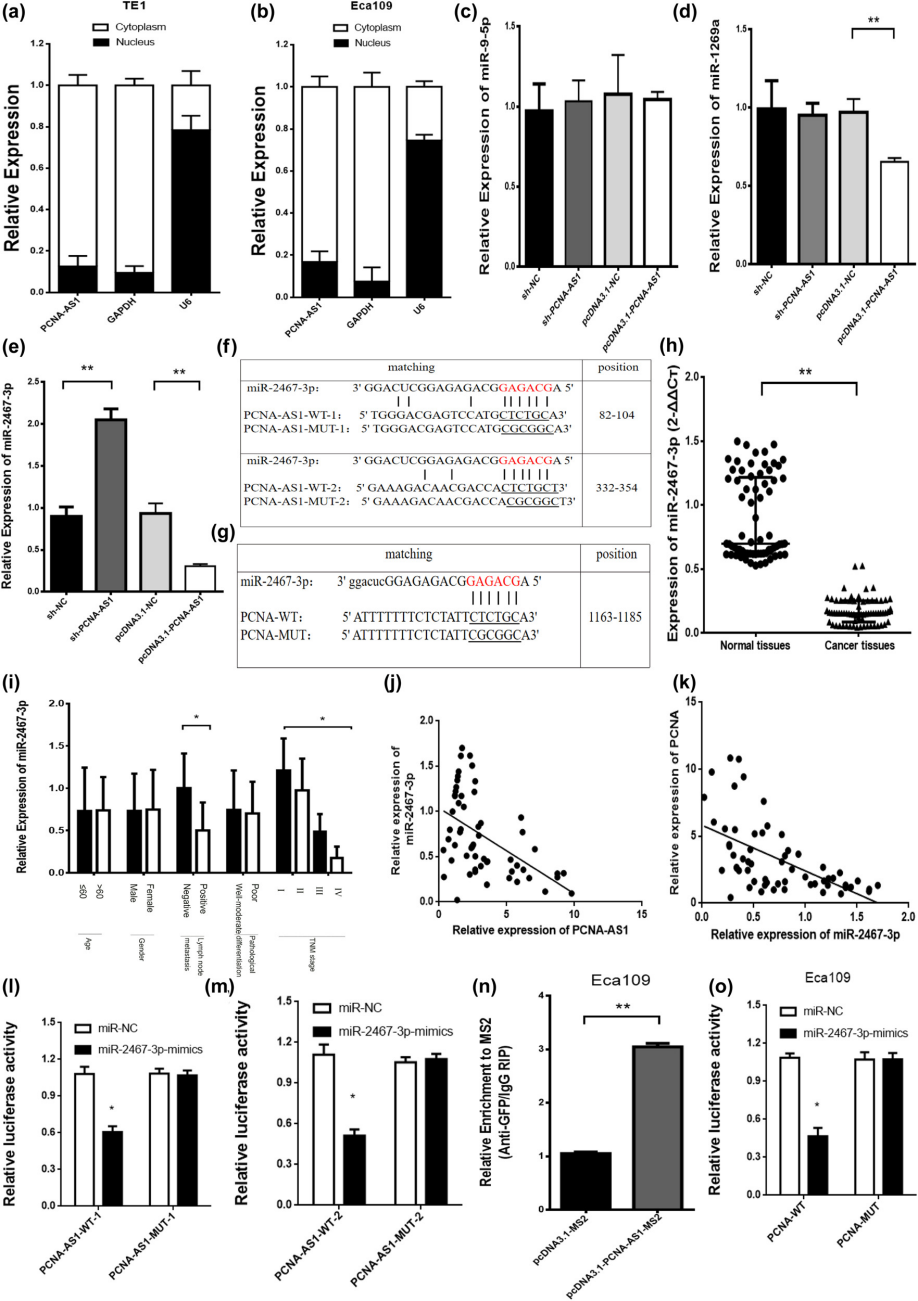
miR-2467-3p includes binding sites to PCNA-AS1/PCNA and is negatively associated with PCNA-AS1/PCNA. (a and b) The distribution of PCNA-AS1 in TE1 and Eca109 cells. (c–e) Effects of PCNA-AS1 on downstream target miRNA. (f and g) The wild-type and mutated (mutant) PCNA-AS1/PCNA binding sites to miR-2467-3p. (h) miR-2467-3p was less expressed in ESCC tissues than in normal tissues. (i) The relative expression of miR-2467-3p was correlated with the TNM stage and lymph node metastasis. (j and k) The correlation of miR-2467-3p and PCNA-AS1 or PCNA. (l and m) Dual luciferase reporter assay to verify the binding activity between PCNA-AS1 and miR-2467-3p. (n) RIP assay showing the high enrichment of PCNA-AS1 by miR-2467-3p. (o) Dual luciferase reporter assay to verify the binding activity between PCNA-AS1 and miR-2467-3p. *P < 0.05 and **P < 0.01.

To examine more closely the role of PCNA-AS1, we explored its distribution in ESCC cells. As shown in Figure 4a and b, PCNA-AS1 was predominantly present in the cytoplasm of TE1 and Eca109 cells. We speculated that PCNA-AS1 acted as a competitive endogenous RNA (ceRNA) to bind specific miRNAs and regulate the expression of target genes such as PCNA.

We performed analyses using bioinformatics databases (miRBase and TargetScan) and found that miR-9-5p, miR-1269a and miR-2467-3p contain potential binding sites for PCNA-AS1 and PCNA 3′-UTR. After the knockdown and overexpression of PCNA-AS1, miR-2467-3p was most remarkably affected by PCNA-AS1 (Figure 4c–e). miR-2467-3p contains two potential binding sites for PCNA-AS1 and a site for binding PCNA. Interestingly, miR-2467-3p could bind PCNA-AS1 and PCNA at the same binding sites (Figure 4f and g). We identified that miR-2467-3p was expressed at low levels in ESCC tissues and was correlated with lymph node metastasis and TNM staging (Figure 4h and i). Meanwhile, miR-2467-3p was negatively correlated with the expression of PCNA-AS1 and PCNA in ESCC tissues (*r* ^*2*^ = 0.266, *r* ^*2*^ = 0.324, *P* < 0.01, respectively, Figure 4j and k).

We next evaluated the regulated relationship between miR-2467-3p and PCNA-AS1. Dual luciferase assay demonstrated that co-transfection of miR-2467-3p mimics with the pmirGLO-PCNA-ASl plasmid in Eca109 cells resulted in decreased relative luciferase activity compared with pmirGLO-PCNA-ASl plasmid alone. However, miR-2467-3p mimics did not affect the luciferase activity driven by pmirGLO-PCNA-ASl-MUT-1, and pmirGLO-PCNA-ASl-MUT-2 contains mutations in the putative binding sites (Figure 4l–m). The RIP assay confirmed that overexpression of PCNA-AS1 enhanced the enrichment of miR-2467-3p (Figure 4n). To further confirm the linkage of miR-2467-3p and PCNA, we generated wild-type or mutant pmirGLO-PCNA vectors and cotransfected them with miR-2467-3p mimics into Eca109 cells. The luciferase activity was obviously reduced in the wild-type group, while no noticeable difference was found in the mutant group (Figure 4o). The outcomes validated miR-2467-3p as a direct target of PCNA-AS1, implying that PCNA-AS1, miR-2467-3p and PCNA may form a ceRNA system to modulate the progression of ESCC.

### 3.5 Biological behavior of miR-2467-3p in ESCC

**Figure 5:**
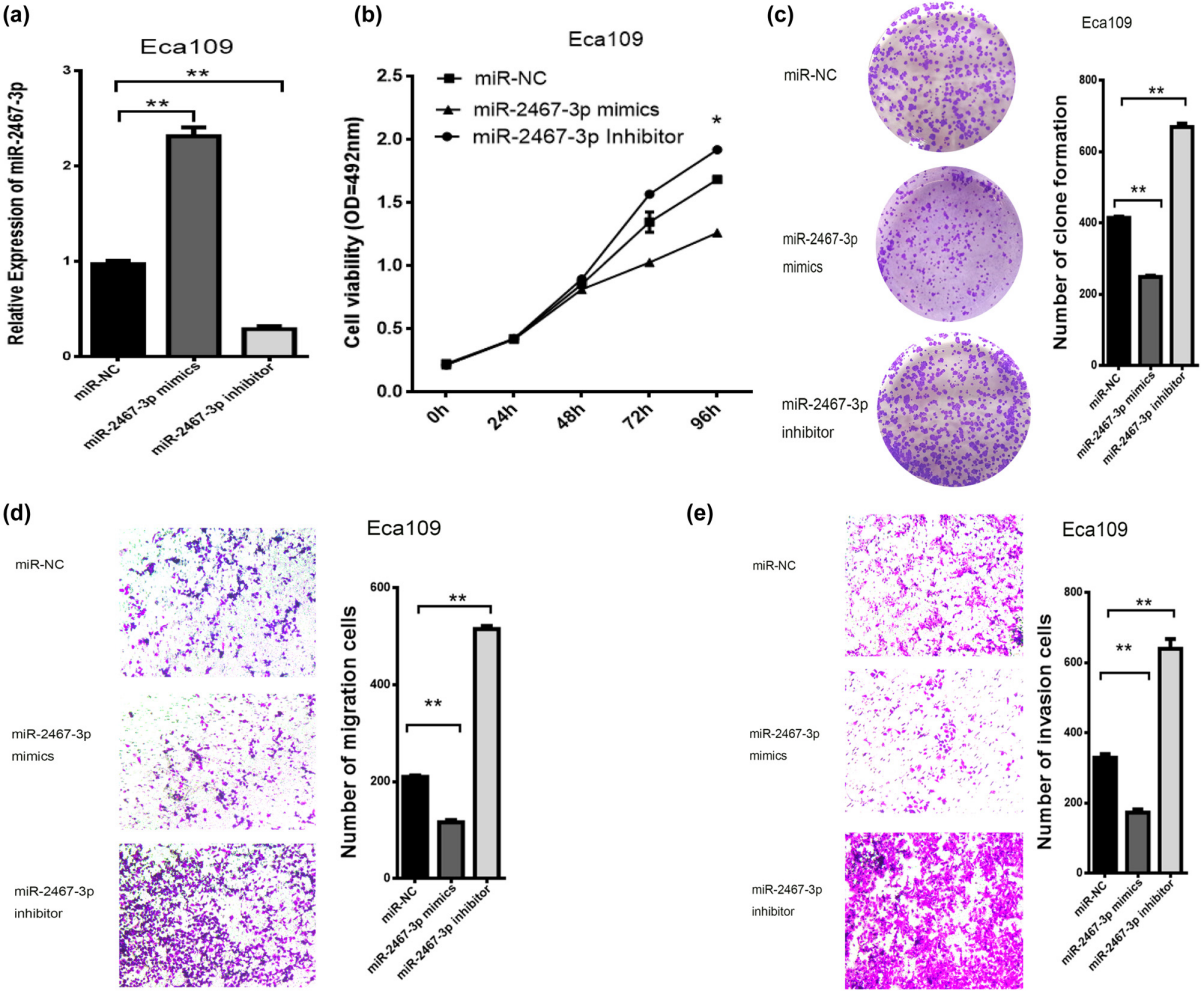
The biological functions of miR-2467-3p in ESCC. (a) After treatment with miR-2467-3p inhibitor or mimics, the transfection efficiency was determined by qRT–PCR. (b–e) The effects of miR-2467-3p mimics and inhibitors on cell viability, clone formation, migration and invasion. *P < 0 .05 and **P < 0.01.

To investigate the function of miR-2467-3p, miR-2467-3p mimics and inhibitors were introduced into Eca109 cells. MTS, clone formation and transwell assay indicated that miR-2467-3p mimics suppressed the proliferation, migration and invasion ability of Eca109 cells, while miR-2467-3p inhibitors produced the opposite effects (Figure 5a–e).

### 3.6 PCNA-AS1 positively regulates PCNA expression and cancer cell activities by controlling miR-2467-3p in ESCC

**Figure 6:**
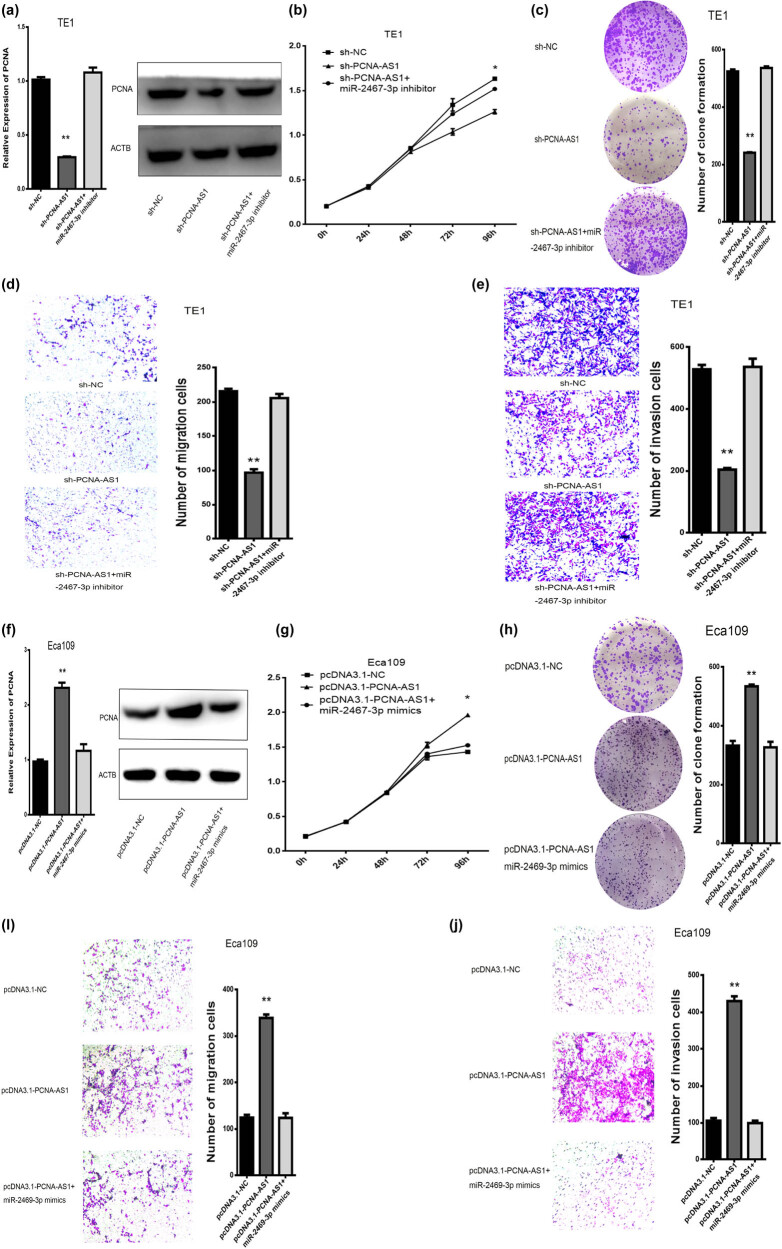
PCNA-AS1 regulates PCNA in a miR-2467-3p manner in ESCC cells. (a–e) The effects of sh-PCNA-AS1 or sh-PCNA-AS1/miR-2467-3p inhibitor co-transfection on the cell viability, migration, clone formation and invasion in the TE1 cells. (f–j) The effects of pcDNA3.1-PCNA-AS1 or pcDNA3.1-PCNA-AS1/miR-2467-3p mimics co-transfection on the cell viability, migration, clone formation and invasion in the TE1 cells. *P < 0.05 and **P < 0.01.

As miR-2467-3p contains binding sites for PCNA-AS1 and PCNA, we hypothesized that PCNA-AS1 adjusts the expression of PCNA through miR-2467-3p. We performed PCNA-AS1 knockdown and miR-2467-3p inhibitor transfection in TE1 cells. In line with our previous results, the knockdown of PCNA-AS1 led to the downregulation of PCNA; however, co-transfection of the miR-2467-3p inhibitor and sh-PCNA-AS1 recovered PCNA expression (Figure 6a), and abolished the biological efficacy of sh-PCNA-AS1 on ESCC cell viability, migration, clone formation and invasion abilities (Figure 6b–e).

We also revealed that while PCNA-AS1 overexpression increased the expression of PCNA, the upregulation of PCNA was markedly reversed by co-transfection with miR-2467-3p mimics (Figure 6f). Co-transfection of the miR-2467-3p mimics and PCNA-AS1 also abolished the effects of PCNA-AS1 on cell proliferation, migration, clone formation and invasion abilities (Figure 6g–j).

## 4 Discussion

Although the mortality rate of ESCC has improved dramatically, the 5-year survivorship of ESCC patients is still low [12]. Consequently, exploring the molecular mechanisms of ESCC is critical to identifying novel biomarkers to enhance the prognosis of ESCC patients [13]. Meanwhile, lncRNAs play an essential role in the molecular mechanism of ESCC [14]. Recent research has indicated that lncRNAs can mediate tumor development by binding to miRNAs as ceRNAs [15]. Zheng and Zhang reported that LINC00963 was increased remarkably in colorectal cancer (CRC) tissues, and knockdown of LINC00963 inhibits the progression of CRC by promoting the expression of miR-124-3p. Moreover, FZD4 restored the inhibition of miR-124-3p on the progression of CRC, indicating that FZD4 may be a directly targeted gene of the LINC00963/miR-124-3p axis in CRC. Thus, LINC00963 represents a promising therapeutic and diagnostic target for CRC treatment [16].

Accumulating evidence has shown that PCNA-AS1 is overexpressed in non-small cell lung cancer [17], CRC [18], gastric cancer [19] and hepatocellular carcinoma [20]. However, the expression and association of PCNA-AS1 and PCNA in ESCC have not been characterized. The function of PCNA-AS1 in ESCC has been unclear. Previous research revealed that PCNA-AS1 acted as an oncogene in ESCC tissues and was correlated with TNM stage and lymph node metastasis. In addition, ESCC patients with high PCNA-AS1 expression have a poorer prognosis than those with low. PCNA-AS1 may function as an oncogenic lncRNA and indicate ESCC diagnosis and prognosis.

PCNA is an acidic non-histone nuclear protein known for its presence in proliferating cells (including normal proliferating cells and cancer cells) [21]. Its molecular weight is 36 kDa and is mainly distributed in the nucleus [22]. It was found that PCNA can directly participate in cell proliferation and DNA synthesis [23,24]. PCNA is also involved in many important cellular events, such as cellular DNA damage repair, cell cycle regulation, chromosome reorganization, DNA methylation and apoptosis [25], and PCNA can act through different pathway-related proteins [26]. PCNA is a key protein for abnormal cell proliferation and is closely related to tumor growth, metastasis and prognosis [27]. It is currently a relatively important class of indicators for studying tumor cell proliferation and assessing the malignant potential of tumors [28]. The specific mechanism is that PCNA expression shows cyclic changes during cell replication, which coincides with changes in DNA content during cell mitosis, and peaks during the mitotic S phase [29,30]. Clinically, PCNA can be used to evaluate tumorigenesis, progression, prognosis and efficacy and thus guide clinical treatment [31]. Studying the correlation of PCNA expression in ESCC is beneficial for understanding the mechanism and biological behavior of ESCC at the molecular level [32].

Natural antisense transcripts can regulate the expression of the sense transcripts in cis or trans [33]. Remarkably, several natural antisense lncRNAs regulate gene expression levels via ceRNAs [34]. In the current study, we discovered that PCNA was positively expressed in ESCC, and its expression was actively associated with the natural antisense transcript of PCNA (PCNA-AS1). Furthermore, PCNA exhibited oncogenic effects in ESCC by promoting ESCC cell proliferation and invasion, which is consistent with previous studies [35]. Natural antisense lncRNAs can regulate the expression of the sense transcript. Chen et al. revealed that the lncRNA ZNF503-AS1 negatively correlates with the expression of ZNF503, and ZNF503-AS1 promotes the differentiation of retinal pigment epithelial cells by downregulating ZNF503 [36]. Shen et al. found that the lncRNA Bhmt-AS1 attenuated liver gluconeogenesis by regulating the expression of Bhmt [37]. Li et al. found that WWTR1 and its antisense lncRNA WWTR1-AS1 were increased in head and neck squamous cell carcinoma, and they were both positively correlated with head and neck squamous cells [38].

LncRNAs exert various functions according to their subcellular localization [39]. PCNA-AS1 was primarily enriched in the cytoplasm, suggesting that PCNA-AS1 may regulate genes at the post-transcriptional level. A few studies have revealed that lncRNAs adjust target genes by competing with miRNAs. Thus, we hypothesized that PCNA-AS1 upregulates the expression of PCNA via a ceRNA mechanism. Using bioinformatics analysis, luciferase analysis and RIP experiments, we investigated the association of PCNA-AS1, miR-2467-3p and PCNA in ESCC. Dual luciferase assays demonstrate that miR-2467-3p downregulated the luciferase activity of pmirGLO-PCNA-AS1; however, the mutated reporter vector did not show this effect. Moreover, the expression of miR-2467-3p was decreased in PCNA-AS1-overexpressing esophageal cancer cells, while miR-2467-3p was upregulated after the PCNA-AS1 interference. RIP analysis displayed that miR-2467-3p was highly enriched in cells with overexpression of PCNA-AS1, suggesting that miR-2467-3p binds directly to PCNA-AS1. PCNA-AS1 serves as a ceRNA to bind miR-2467-3p, thereby reducing the level of free miR-2467-3p in the cytoplasm. Furthermore, dual luciferase and western blot assay revealed that PCNA was a directly targeted gene of miR-2467-3p.

Previous reports have shown that miR-2467-3p serves a tumor suppressor function in different tumors [40,41,42]. Our findings indicated that miR-2467-3p suppresses the invasion and metastasis of ESCC cells, suggesting that miR-2467-3p also exhibits a tumor suppressor effect in ESCC. Rescue experiments further validated PCNA-AS1 as a ceRNA adsorbing miR-2467-3p, promoting PCNA expression and esophageal cancer progression.

In conclusion, these outcomes revealed that PCNA-AS1 was upregulated in ESCC and competitively bound miR-2467-3p as a ceRNA to promote PCNA expression. PCNA-AS1 might be a possible target for ESCC treatment, and this thesis offered new insights into the study of ESCC.

## Supplementary Material

Supplementary Table
