# Supplementary material for: Antisense lncRNA PCNA-AS1 promotes esophageal squamous cell carcinoma progression through the miR-2467-3p/PCNA axis
Source: Open Med (Wars). 2022 Sep 20;17(1):1483–94. doi: 10.1515/med-2022-0552 (PMC9490863; doi:10.1515/med-2022-0552)
Supplement: Supplementary Table [file med-2022-0552-sm.pdf]

# Supplementary material

**Table S1:** Clinicopathologic characteristics of patients with ESCC

| | Group | N (%) |
|---|---|---|
| Gender | | |
| | Male | 47 (78.3) |
| | Female | 13 (21.7) |
| Age | | |
| | ≤60 | 25 (41.7) |
| | >60 | 35 (58.3) |
| Histological grade | | |
| | Low | 14 (23.3) |
| | Middle or high | 46 (76.7) |
| Lymph node metastasis | | |
| | Negative (N0) | 28 (46.7) |
| | Positive (N1/N2/N3) | 32 (53.3) |
| TNM stage | | |
| | I | 7 (11.7) |
| | II | 25 (41.6) |
| | III | 23 (38.3) |
| | IV | 5 (8.4) |

**Table S2:** Primers used for reverse transcription and qPCR

| **Names** | **Sequences** |
|---|---|
| Reverse Transcription | |
| Has-miR-26a-5p stem-loop | 5′-GTCGTATCCAGTGCAGGGTCCGAGGTATTCGCACTGGATACGACAGCCTATC-3′ |
| Hsa-miR-211-5p stem-loop | 5′-GTCGTATCCAGTGCAGGGTCCGAGGTATTCGCACTGGATACGACAGGCGAAG -3′ |
| Hsa-miR-1297 stem-loop | 5′-GTCGTATCCAGTGCAGGGTCCGAGGTATTCGCACTGGATACGACCACCTGAA-3′ |
| Hsa-miR-9-5p stem-loop | 5′-GTCGTATCCAGTGCAGGGTCCGAGGTATTCGCACTGGATACGACTCATACAG -3′ |
| Hsa-miR-1269a stem-loop | 5′-GTCGTATCCAGTGCAGGGTCCGAGGTATTCGCACTGGATACGACCCAGTAGC -3′ |
| Hsa-miR-2467-3p stem-loop | 5′-GTCGTATCCAGTGCAGGGTCCGAGGTATTCGCACTGGATACGACCCTGAGCC -3′ |
| U6 stem-loop | 5′-AACGCTTCACGAATTTGCGT-3′ |
| **qRT-PCR** | |
| PCNA-AS1 | F:5′-GGCGGGAAGGAGGAAAGTC-3′ |
| | R:5′-GGTTGCAGGCGTAGCAGAG-3′ |
| PCNA | F:5′-CAACGGTGACACTCAGTAT-3′ |
| | R:5′-TGGCATCTTAGAAGCAGTT-3′ |
| miR-26a-5p | F:5′-GTGCAGGGTCCGAGGTATT-3′ |
| | R:5′-GCCGTTCAAGTAATCCAGGATAGG-3′ |
| miR-211-5p | F:5′-GTGCAGGGTCCGAGGTATT-3′ |
| | R:5′-GCCGTTCCCTTTGTCATCCTTCGC-3′ |
| miR-1297 | F:5′-GTGCAGGGTCCGAGGTATT-3′ |
| | R:5′-GCCGTTCAAGTAATTCAGG-3′ |
| miR-9-5p | F:5′-GTGCAGGGTCCGAGGTATT-3′ |
| | R:5′-GCCGTCTTTGGTTATCTAGCTGTAT-3′ |
| miR-1269a | F:5′-GTGCAGGGTCCGAGGTATT-3′ |
| | R:5′-GCCGCTGGACTGAGCCGTGCTACT-3′ |
| miR-2467-3p | F:5′-GTGCAGGGTCCGAGGTATT-3′ |
| | R:5′-GCCGCTGGACTGAGCCGTGCTACT-3′ |
| U6 | F:5′-CTCGCTTCGGCAGCACA-3′ |
| | R:5′-AACGCTTCACGAATTTGCGT-3′ |
| GAPDH | F:5′-AGGTGAAGGTCGGAGTCAACG-3′ |
| | R:5′-AGGGGTCATTGATGGCAACA-3′ |
| ACTB | F:5′-ACCGAGCGCGGCTACAG-3′ |
| | R:5′-CTTAATGTCACGCACGATTTCC-3′ |

F:Forward primer; R:Reverse primer.

**Table S3:** Primers used for shRNAs and microRNA sequences

| Name | Sequences |
|---|---|
| PCNA-AS1-Homo-363 | F:5′-AACCGTTTAATGCCGCCGCGT-3′ |
| Hsa-miR-2467-3p mimics | F:5′-AGCAGAGGCAGAGAGGCUCAGG-3′ |

F:Forward primer; R:Reverse primer.

**Table S4:** Primers used for vectors construction

| Names | Sequences |
|---|---|
| pmirGLO-PCNA-AS1-1 | F:5′-CCGGAATCCTGAGCTGCACCAAAGAGACGT-3′<br>R:5′-GCTCTAGAGTGTTGGAGGCACTCAAGGAC-3′ |
| pmirGLO-PCNA-AS1-2 | F:5′-CCGGAATCCGGAAAGTCTAGCTGGTTTCGG-3′<br>R:5′-GCTCTAGACGTAGCAGAGTGGTCGTTGTC-3′ |
| pmirGLO-PCNA-AS1-1(mut) | F:5′-GGCAGGTTTACACCGCTGGAG-3′<br>R:5′-GCGCATGGACTCGTCCCACGT-3′ |
| pmirGLO-PCNA-AS1-2(mut) | F:5′-GGCTACGCCTGCAACCGTTTA-3′<br>R:5′-GCGTGGTCGTTGTCTTTCTAGG-3′ |
| pmirGLO-PCNA | F:5′-CCGGAATCCACTGAAGTCTTTTCTGTCACCA-3′<br>R:5′-GCTCTAGAGACCAGATCTGACTTTGGACT-3′ |
| pmirGLO-PCNA (mut) | F:5′-GGCAATTTGTTTAAAGAATAAAGTCCAAAG-3′<br>R:5′-GCGAATAGAGAAAAAAATAGGTTATTTAC-3′ |

F:Forward primer; R:Reverse primer.
